# Modulating Oxidative Stress and Inflammatory Responses of *Aloe vera*–Silk Fibroin–Chitosan Gel Formulation in Macrophages and Diabetic Fibroblasts Under Chronic Inflammatory-like Conditions

**DOI:** 10.3390/gels12080662

**Published:** 2026-07-23

**Authors:** Phassorn Khumfu, Vladimir Spasovski, Sukunya Ross, Gareth Ross, Céline Viennet, Jarupa Viyoch

**Affiliations:** 1 Department of Pharmaceutical Technology, Faculty of Pharmaceutical Sciences, Naresuan University, Phitsanulok 65000, Thailand; phassornk66@nu.ac.th; 2 UMR 1098 RIGHT INSERM EFS FC, DImaCell Imaging Resource Center, Marie & Louis Pasteur University, 25000 Besançon, France; vladimir.spasovski@umlp.fr; 3 Department of Chemistry, Faculty of Science, Naresuan University, Phitsanulok 65000, Thailand; sukunyaj@nu.ac.th (S.R.); gareth@nu.ac.th (G.R.); 4 Center of Excellence for Innovation in Chemistry (PERCH–CIC), Faculty of Pharmaceutical Sciences, Naresuan University, Phitsanulok 65000, Thailand

**Keywords:** silk fibroin, *Aloe vera* gel extract, thermosensitive gel, diabetic wound healing, anti-inflammation, macrophages, fibroblasts

## Abstract

Chronic and diabetic wounds are characterized by persistent inflammation, excessive oxidative stress, and impaired cellular responses, collectively leading to delayed tissue repair. This study aimed to evaluate a thermoresponsive gel incorporating *Aloe vera* (AV) extracts and silk fibroin (SF), focusing on its biocompatibility, anti-inflammatory, antioxidant, and pro-regenerative potential. The AV: SF mixture and gel formulation were evaluated in macrophage-like THP-1 cells, and human diabetic dermal fibroblasts (HDdF) under non-induced and LPS/IL-6/TNF-α-induced inflammatory conditions. Both formulations maintained high cell viability across all cell types, indicating good biocompatibility. Under inflammatory conditions, both the AV: SF mixture and gel significantly reduced intracellular ROS levels and suppressed TNF-α and IL-1β secretion. In addition, NF-κB p65 activation was attenuated in both macrophage-like THP-1 cells and HDdF. Macrophage-like THP-1 cells displayed the M2 (CD80^low^/CD206^high^) phenotype, indicating modulation of macrophage polarization toward wound remodeling and repair. Overall, the AV: SF-based gel system demonstrated coordinated antioxidant, anti-inflammatory, and pro-regenerative effects in vitro. These findings support its potential as a multifunctional biomaterial for modulating inflammation-associated cellular dysfunction in chronic and diabetic wound environments.

## 1. Introduction

Diabetic ulcers remain a major clinical challenge because persistent inflammation, impaired fibroblast function, and defective extracellular matrix (ECM) remodeling collectively delay wound healing and increase the risks of infection, chronic tissue damage, and lower-limb amputation [1,2,3]. Although current wound care strategies improve wound management, they often fail to address the complex cellular and molecular abnormalities underlying chronic diabetic wounds.

Macrophages and fibroblasts are key regulators of wound healing. Macrophages orchestrate inflammatory responses and coordinate the transition from inflammation to tissue repair, whereas fibroblasts are responsible for ECM synthesis, collagen deposition, and tissue remodeling [4]. Under diabetic conditions, prolonged macrophage activation and impaired fibroblast function disrupt this coordinated healing process, resulting in persistent inflammation and delayed tissue regeneration [3,4,5,6]. Consequently, therapeutic approaches capable of simultaneously regulating inflammation and promoting tissue repair are highly desirable.

Biomaterial-based gels have emerged as promising wound dressings because of their high-water content, biocompatibility, and ability to mimic the native extracellular matrix while maintaining a moist wound environment [7,8]. Recent advances have focused on multifunctional biomaterials that combine several therapeutic functions within a single platform, including antioxidant, anti-inflammatory, antimicrobial, angiogenic, and extracellular matrix-supporting activities, thereby addressing multiple pathological processes involved in chronic diabetic wounds [9,10,11].

Among naturally derived biomaterials, silk fibroin (SF) has attracted considerable attention because of its excellent mechanical properties, biodegradability, and ability to support cell adhesion, proliferation, and tissue regeneration [12,13,14,15]. *Aloe vera* (Aloe barbadensis Miller) contains diverse bioactive polysaccharides, glycoproteins, and phenolic compounds that exhibit antioxidant, anti-inflammatory, and regenerative activities by promoting fibroblast proliferation, collagen synthesis, re-epithelialization, and modulation of inflammatory responses [16,17]. Chitosan (CS) further complements these properties through its intrinsic antibacterial activity, hemostatic capacity, biocompatibility, and excellent gel-forming ability, making it an attractive component for advanced wound dressings [18,19,20,21,22].

Previous studies have demonstrated that silk fibroin/*Aloe vera* biomaterials accelerate diabetic wound healing by enhancing fibroblast attachment, proliferation, collagen deposition, and wound closure in streptozotocin-induced diabetic animal models [23]. However, these studies primarily evaluated wound-healing outcomes without investigating the cellular and molecular mechanisms responsible for their therapeutic effects. In particular, the immunomodulatory interactions between inflammatory macrophages and diabetic fibroblasts within the chronic wound microenvironment remain poorly understood [23,24,25,26,27]. Because persistent inflammation is a major contributor to impaired diabetic wound healing, elucidating these mechanisms is essential for the rational design of next-generation bioactive wound care products.

To address this knowledge gap, *Aloe vera* extract (AV), silk fibroin (SF), and chitosan (CS) were incorporated into a thermoresponsive gel designed to provide both structural support and biological regulation of the wound microenvironment. Unlike previously reported fibroin/*Aloe vera* systems that mainly emphasized fibroblast responses and wound closure [23,24,25,26,27], the present study focused on the immunomodulatory properties of the composite gel under diabetic inflammatory conditions. The thermoresponsive characteristics of the gel further facilitate in situ application, intimate contact with the wound surface, and prolonged retention of bioactive components.

To better reproduce the inflammatory microenvironment of chronic diabetic wounds, an in vitro inflammatory model was established using lipopolysaccharide (LPS), interleukin-6 (IL-6), and tumor necrosis factor-alpha (TNF-α), representing bacterial stimulation together with key inflammatory cytokines associated with persistent diabetic inflammation [23,28,29,30,31]. This model provides a biologically relevant platform for evaluating the antioxidant, anti-inflammatory, and regenerative activities of multifunctional wound biomaterials [32,33,34,35].

Therefore, this study systematically investigated the biological activities of an AV–SF–CS thermoresponsive gel using macrophage-like THP-1 cells and human diabetic dermal fibroblasts (HDdFs) under normal and inflammatory conditions. Specifically, cytocompatibility, fibroblast proliferation, intracellular reactive oxygen species (ROS), inflammatory cytokine secretion, NF-κB signaling, and macrophage polarization were evaluated to elucidate the mechanisms through which the thermoresponsive gel modulates the diabetic wound microenvironment. These findings provide mechanistic insights into the immunomodulatory and regenerative potential of natural polymer-based biomaterials and support the development of next-generation bioactive wound dressings for chronic diabetic ulcers.

## 2. Results

### 2.1. Cytotoxicity of the Gel Extract to Macrophage-like THP-1 and HDdF

**Figure 1:**
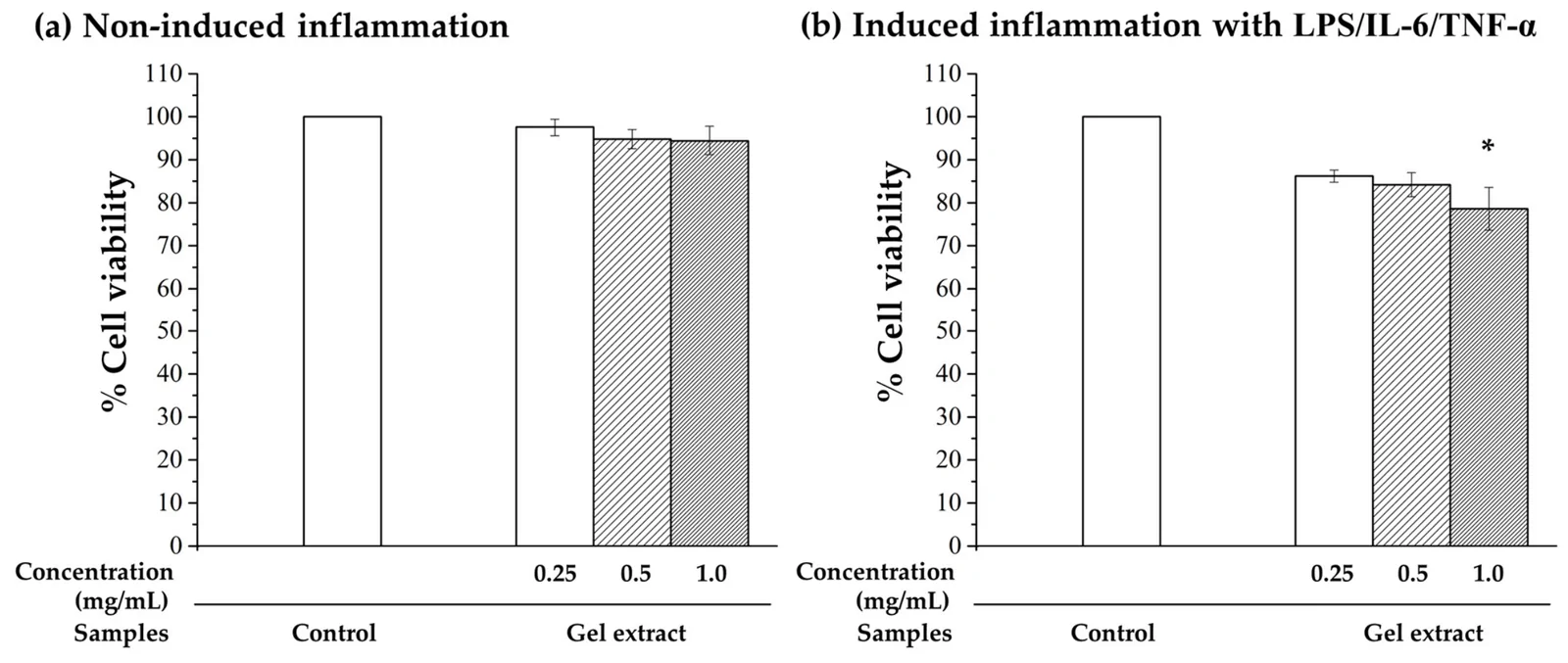
Percentage of cell viability of (**a**) macrophage-like THP-1 cells and (**b**) macrophage-like THP-1 cells under induced inflammatory conditions, treated with gel at concentrations of 0.25, 0.5, and 1.0 mg/mL, as determined by the MTT assay. Statistical significance was assessed using one-way ANOVA followed by Fisher’s LSD post hoc test. An asterisk indicates a significant difference between groups (* *p* < 0.05) compared with the induced inflammation group.

**Figure 2:**
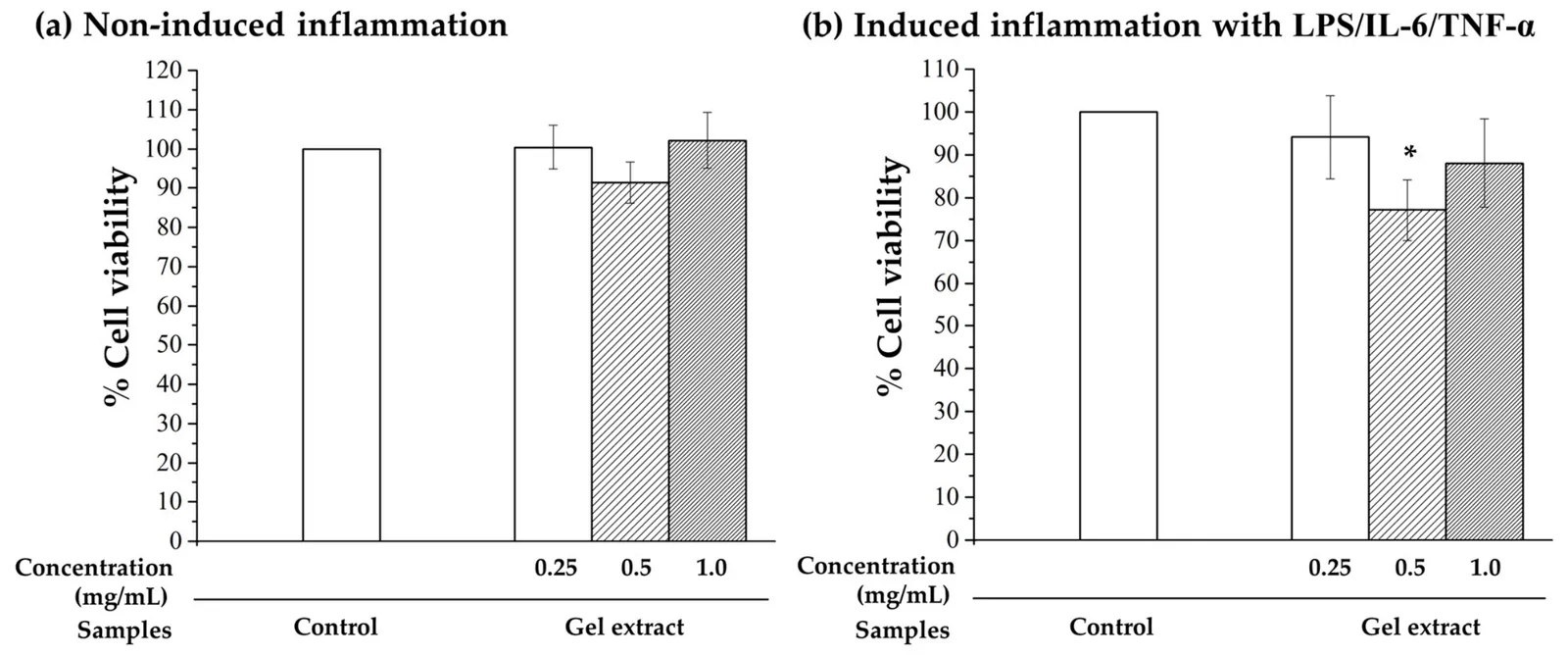
Percentage of cell viability of (**a**) HDdF cells and (**b**) HDdF cells under induced inflammatory conditions, treated with gel at concentrations of 0.25, 0.5, and 1.0 mg/mL, as determined by the MTT assay. Statistical significance was assessed using one-way ANOVA followed by Fisher’s LSD post hoc test. An asterisk indicates a significant difference between groups (* *p* < 0.05) compared with the induced inflammation group.

The cytocompatibility of the gel extract was evaluated using the MTT assay in macrophage-like THP-1 cells and human diabetic dermal fibroblasts (HDdF) under both normal and inflammatory conditions. The inflammatory model was established using lipopolysaccharide (LPS), interleukin-6 (IL-6), and tumor necrosis factor-alpha (TNF-α) to mimic the pro-inflammatory microenvironment associated with chronic diabetic wounds [28,29,30,31]. The gel extract was tested at concentrations of 0.25, 0.5, and 1.0 mg/mL based on our previous study [24], and the corresponding cell viability results are presented in Figure 1 and Figure 2.

Under normal culture conditions, the gel extract exhibited good cytocompatibility in both cell types. Cell viability remained above 80% at all tested concentrations in macrophage-like THP-1 cells (Figure 1a) and HDdF (Figure 2a), indicating that the gel extract was non-cytotoxic according to ISO 10993-5 criteria (cell viability ≥ 70%) [36].

Following inflammatory stimulation, both THP-1 cells (Figure 1b) and HDdF (Figure 2b) exhibited a concentration-dependent decrease in cell viability. The highest viability was observed at 0.25 mg/mL, followed by a slight reduction at 0.5 mg/mL and a more pronounced decline at 1.0 mg/mL, with viability decreasing to approximately 75% under inflammatory conditions. Despite this statistically significant reduction, cell viability remained above the accepted cytotoxicity threshold of 70%, indicating that the gel extract retained acceptable cytocompatibility even under inflammatory stress.

The favorable cytocompatibility observed under inflammatory conditions may be explained by the complementary biological functions of the gel components. Silk fibroin provides an extracellular matrix-like substrate that supports cell survival and proliferation [12,15,17,37], whereas *Aloe vera* supplies bioactive polysaccharides and phenolic compounds known to promote tissue regeneration, attenuate inflammation, and maintain cellular viability [10,11,16,17,18,37]. Chitosan further enhances cellular compatibility while contributing to the structural integrity and stability of the thermoresponsive gel system [20,21,24,26,27]. The modest reduction in cell viability at the highest concentration is likely attributable to increased cellular sensitivity caused by oxidative stress and inflammatory signaling rather than to the intrinsic cytotoxicity of the gel. Consistent with previous reports, inflammatory mediators such as LPS, TNF-α, and IL-6 increase intracellular oxidative stress and metabolic burden, thereby reducing mitochondrial metabolic activity measured by the MTT assay [5,6,28,29,30,31,34]. Nevertheless, maintenance of cell viability above 70% demonstrates that the AV: SF thermoresponsive gel preserves cellular compatibility within a diabetic wound-like inflammatory microenvironment.

Considering its acceptable cytocompatibility together with its potential to deliver higher levels of bioactive compounds, the concentration of 1.0 mg/mL was selected for subsequent in vitro investigations of the antioxidant, anti-inflammatory, and immunomodulatory activities of the gel.

### 2.2. Proliferation of Human Diabetic Dermal Fibroblasts

**Figure 3:**
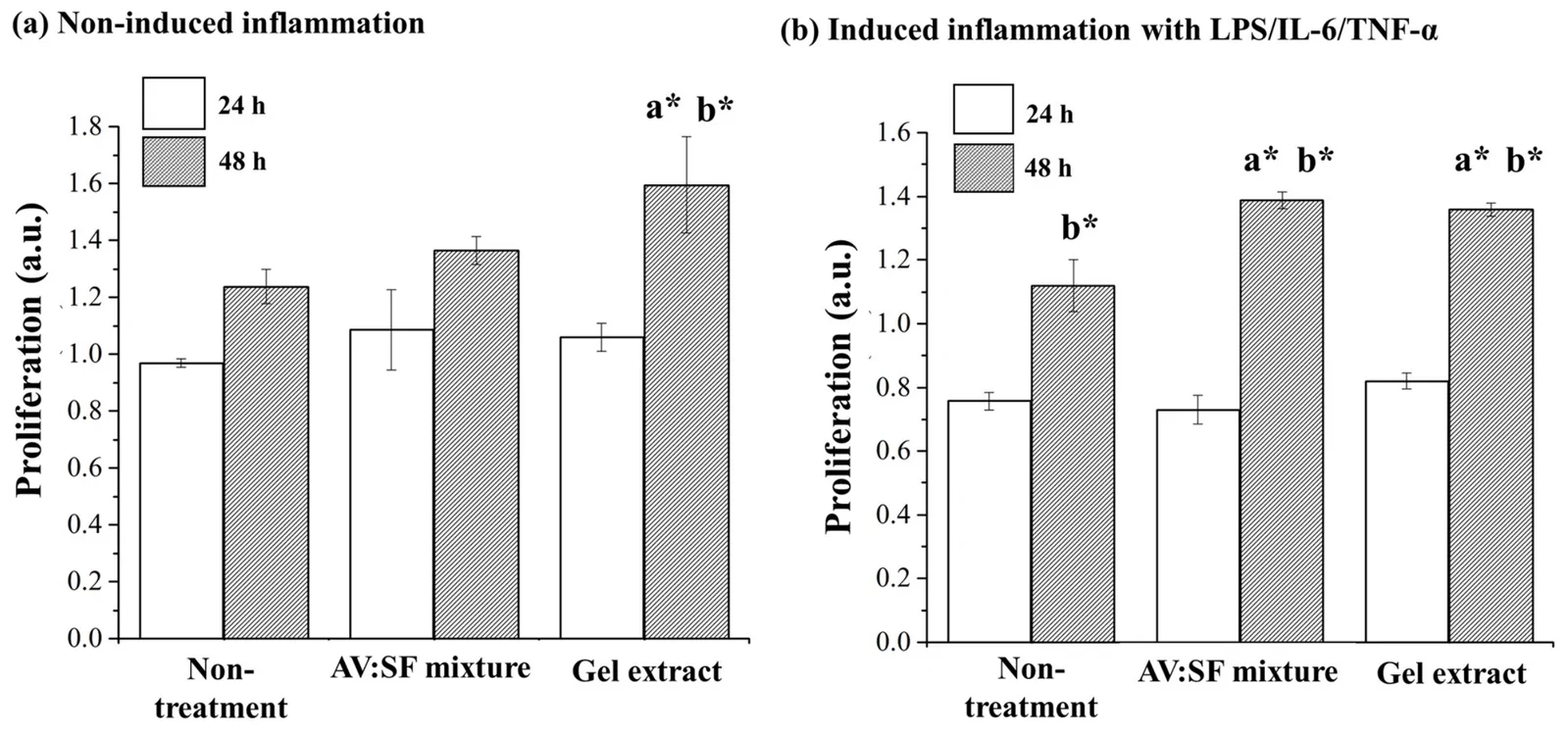
Cell proliferation of human diabetic dermal fibroblasts (HDdF) treated with AV: SF mixture and gel extract at 24 h and 48 h under (**a**) normal and (**b**) inflammatory conditions. Inflammatory conditions were induced using LPS, IL-6, and TNF-α to mimic a chronic wound microenvironment. Statistical significance was determined by one-way ANOVA followed by Fisher’s LSD post hoc test. a indicates statistically significant differences (* *p* < 0.05) between treated and non-treated (control) groups at the same time point, while b indicates statistically significant differences (* *p* < 0.05) between 24 h and 48 h within the same treatment condition.

The proliferative effects of the AV: SF mixture and gel formulation were evaluated in human diabetic dermal fibroblasts (HDdF) under normal and inflammatory conditions. An in vitro inflammatory model was established using lipopolysaccharide (LPS), interleukin-6 (IL-6), and tumor necrosis factor-alpha (TNF-α) to mimic the pro-inflammatory microenvironment associated with chronic diabetic wounds [28,29,30,31]. Cell proliferation was assessed after 24 and 48 h of treatment, and the results are presented in Figure 3.

After 24 h of treatment, both the AV: SF mixture and gel formulation produced a slight increase in fibroblast proliferation compared with the non-treated control; however, the differences were not statistically significant under either normal or inflammatory conditions. By 48 h, both treatments significantly enhanced fibroblast proliferation relative to the non-treated control, with a markedly greater response than that observed at 24 h, demonstrating a clear time-dependent effect. The gel formulation consistently exhibited a stronger proliferative response than the AV: SF mixture, particularly under inflammatory conditions, suggesting that the thermoresponsive gel matrix enhances the regenerative activity of the incorporated bioactive components.

The enhanced fibroblast proliferation is consistent with the complementary biological activities of the gel components. *Aloe vera* contains bioactive polysaccharides, glycoproteins, and phenolic compounds that promote fibroblast proliferation, collagen synthesis, and extracellular matrix production [10,11,16,17,18,37], whereas silk fibroin provides an extracellular matrix-like substrate that supports cell attachment, survival, and proliferation [9,12,15,17,23,37]. Chitosan further contributes by forming a biocompatible three-dimensional network that stabilizes the gel structure and enables sustained exposure of fibroblasts to bioactive molecules, thereby facilitating cellular activities associated with tissue regeneration [19,20,21,22,26,27].

The greater proliferative activity observed after 48 h indicates that the regenerative effects of the AV: SF system require sufficient exposure time to activate cellular responses rather than inducing an immediate mitogenic effect. This time-dependent behavior is consistent with previous studies demonstrating that natural polymer-based biomaterials progressively stimulate fibroblast proliferation through modulation of cell–matrix interactions, sustained bioactive release, and growth factor-related signaling pathways [3,7,8,9,17,23,25,26,27]. Furthermore, oxidative stress and persistent inflammatory cytokines are known to impair fibroblast proliferation under diabetic conditions [5,6,29], suggesting that the enhanced proliferative response observed following treatment may also be associated with attenuation of the inflammatory microenvironment. Collectively, these findings suggest that the AV: SF thermoresponsive gel supports fibroblast-mediated tissue regeneration under inflammatory conditions and may therefore contribute to improved healing of chronic diabetic wounds.

### 2.3. Intracellular ROS Levels in Macrophage-like THP-1 Cells and HDdF

Intracellular reactive oxygen species (ROS) generation was evaluated in macrophage-like THP-1 cells and human diabetic dermal fibroblasts (HDdF) under normal and inflammatory conditions using the 2′,7′-dichlorofluorescin diacetate (DCFH-DA) fluorescent probe. To determine whether the observed biological activity primarily originated from the major bioactive components, *Aloe vera* and silk fibroin, the AV: SF mixture (1:36), corresponding to the composition of the thermoresponsive gel, was evaluated alongside the gel extract. Based on the cytocompatibility results, the gel extract was tested at 1.0 mg/mL, while the AV: SF mixture was examined at an equivalent concentration.

**Figure 4:**
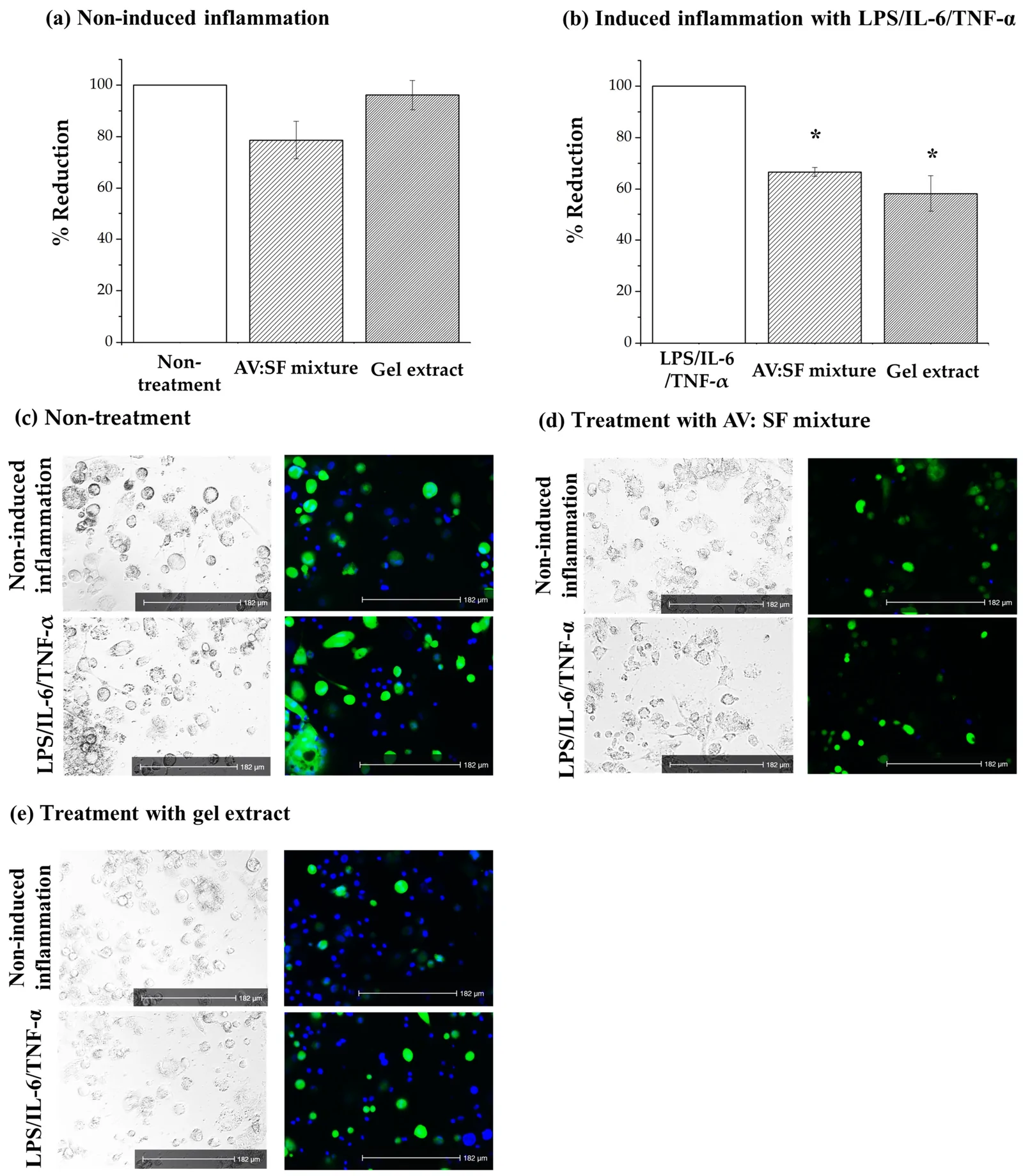
Intracellular reactive oxygen species (ROS) fluorescence intensity and representative fluorescence images of macrophage-like THP-1 cells: (**a**) non-induced inflammation, (**b**) induced inflammatory conditions with LPS/IL-6/TNF-α. (**b**–**e**) Fluorescence images show green signals representing ROS generation and blue signals indicating DAPI-stained nuclei. Statistical significance was assessed using one-way ANOVA followed by Fisher’s LSD post hoc test. An asterisk indicates statistically significant differences (* *p* < 0.05) between treatment and non-treatment groups.

**Figure 5:**
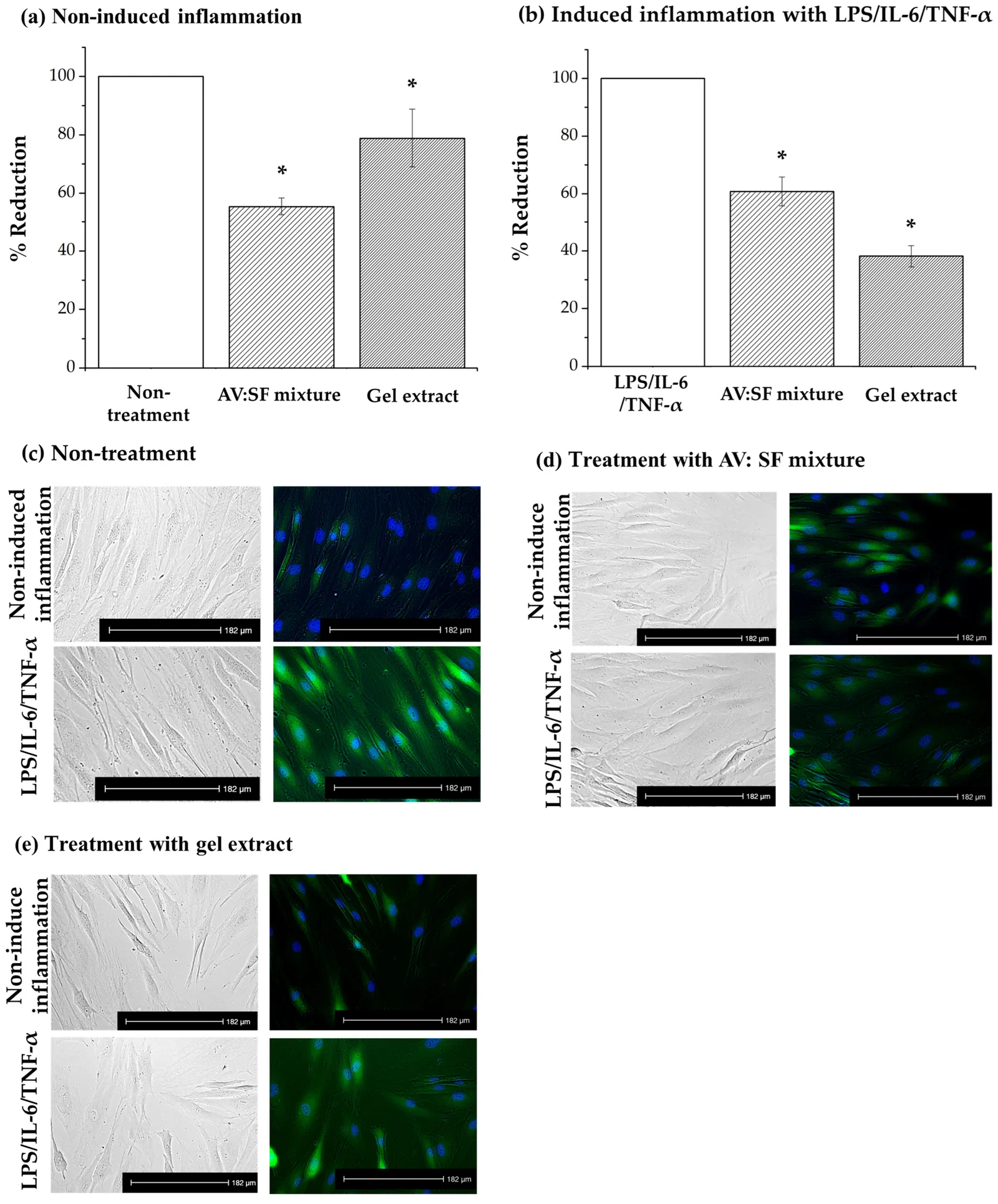
Intracellular reactive oxygen species (ROS) fluorescence intensity and representative fluorescence images of human diabetic dermal fibroblasts (HDdF): (**a**) non-induced inflammation, (**b**) induced inflammatory conditions with LPS/IL-6/TNF-α. (**b**–**e**) Fluorescence images show green signals representing ROS generation and blue signals indicating DAPI-stained nuclei. Statistical significance was assessed using one-way ANOVA followed by Fisher’s LSD post hoc test. An asterisk indicates statistically significant differences (* *p* < 0.05) between treatment and non-treatment groups.

Under non-induced conditions, both THP-1 cells (Figure 4a) and HDdF (Figure 5a) exhibited low basal intracellular ROS levels, and treatment with either the AV: SF mixture or the thermoresponsive gel did not significantly affect ROS production. Following stimulation with lipopolysaccharide (LPS), interleukin-6 (IL-6), and tumor necrosis factor-alpha (TNF-α), intracellular ROS levels markedly increased in macrophage-like THP-1 cells compared with the non-induced control (Figure 4b), confirming successful induction of oxidative stress [28,29,30,31]. In contrast, HDdF exhibited only a slight, non-significant increase in ROS following inflammatory stimulation (Figure 5b), indicating a lower oxidative response than macrophages. Treatment with both the AV: SF mixture and the thermoresponsive gel significantly reduced intracellular ROS levels in inflammatory THP-1 cells and HDdF compared with the untreated inflammatory controls (*p* < 0.05). The gel formulation consistently produced a greater reduction in ROS than the AV: SF mixture, suggesting that incorporation into the thermoresponsive matrix enhances antioxidant efficacy.

The pronounced ROS production observed in inflammatory THP-1 cells reflects the physiological role of activated macrophages, which generate reactive oxygen species as part of the innate immune response. However, excessive ROS accumulation contributes to persistent oxidative stress, prolonged inflammation, and delayed wound healing, particularly in diabetic ulcers [1,4,5,32,33,34,35]. Although fibroblasts generated substantially lower ROS levels following inflammatory stimulation, oxidative stress can still impair fibroblast proliferation, collagen synthesis, and extracellular matrix remodeling, thereby limiting tissue repair [3,5,6].

The reduction in intracellular ROS is consistent with the complementary antioxidant properties of the gel components. *Aloe vera* contains antioxidant polysaccharides, flavonoids, and phenolic compounds that scavenge reactive oxygen species and attenuate oxidative damage [10,16,17,18,37], whereas silk fibroin has been reported to maintain cellular homeostasis and improve cellular resistance to oxidative stress while supporting tissue regeneration [9,12,15,17,23,37]. Chitosan may further contribute through its intrinsic antioxidant properties and by stabilizing the thermoresponsive gel matrix, thereby improving the retention and sustained availability of the incorporated bioactive compounds [19,20,21,22,26,27]. These characteristics likely explain the stronger antioxidant effect observed for the gel formulation compared with the AV: SF mixture.

Collectively, these findings demonstrate that the AV: SF thermoresponsive gel effectively attenuates intracellular oxidative stress under inflammatory conditions while preserving physiological ROS levels under basal conditions. This selective antioxidant activity provides a mechanistic basis for the subsequent suppression of inflammatory signaling and supports tissue regeneration during chronic diabetic wound healing.

### 2.4. IL-1β and TNF-α Secretion

The anti-inflammatory effects of the AV: SF mixture and gel extract were evaluated by measuring the secretion of the pro-inflammatory cytokines TNF-α and IL-1β in macrophage-like THP-1 cells and human diabetic dermal fibroblasts (HDdF) under both non-induced and inflammatory conditions. An in vitro inflammatory model was established using lipopolysaccharide (LPS), interleukin-6 (IL-6), and tumor necrosis factor-alpha (TNF-α) to reproduce the pro-inflammatory microenvironment associated with chronic diabetic wounds [28,29,30,31]. Although cytokine secretion was not normalized to cell number or total protein, all experimental groups were evaluated under identical experimental conditions. Therefore, the consistent reduction in cytokine levels despite increased cell proliferation suggests that the observed effects primarily reflect treatment-induced modulation of inflammatory activity rather than differences in cell numbers.

**Figure 6:**
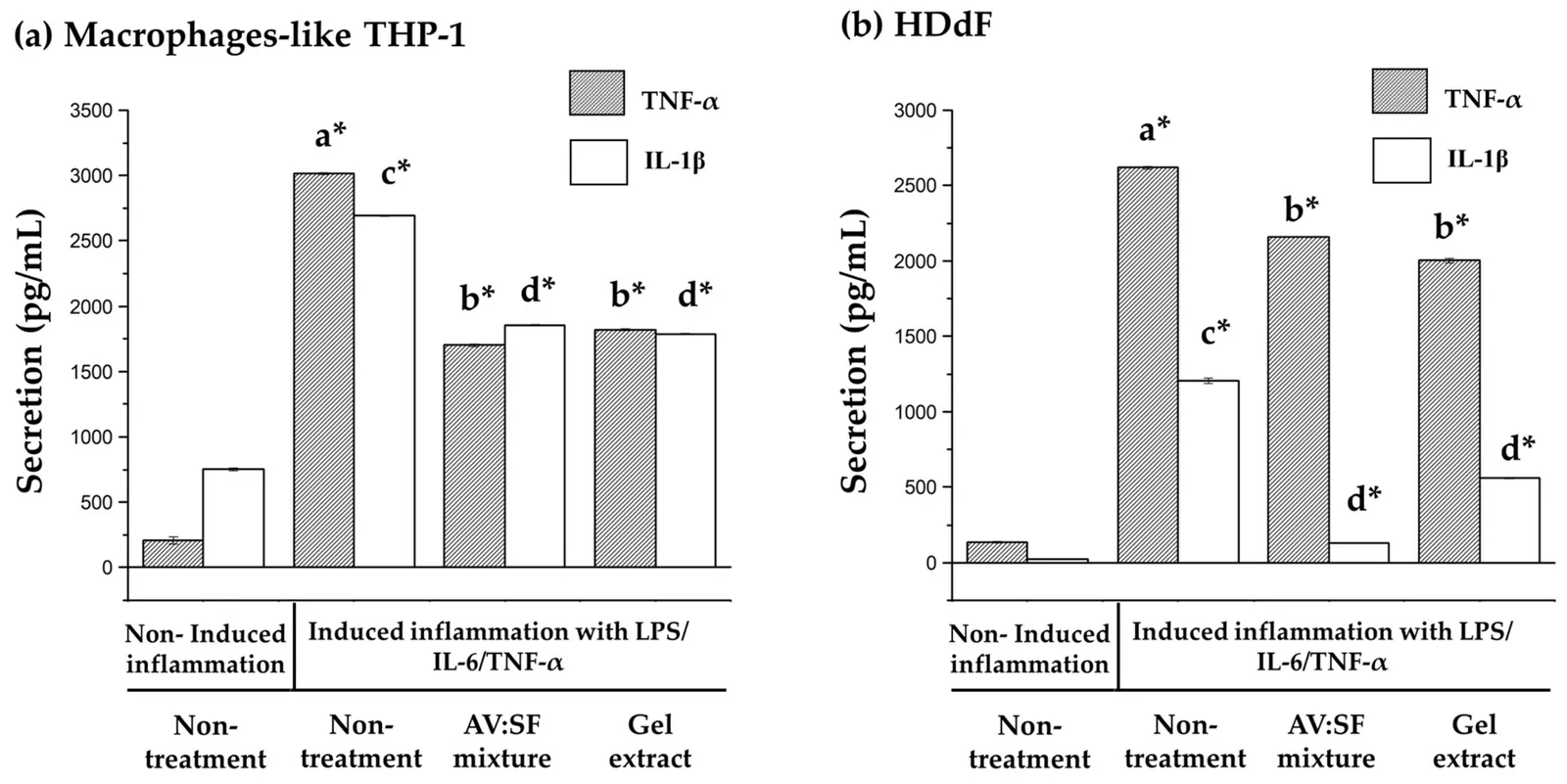
Effects of the AV: SF mixture and gel extract (1:36) on pro-inflammatory cytokine secretion in (**a**) macrophage-like THP-1 cells and (**b**) human diabetic dermal fibroblasts (HDdF) under non-induced and inflammatory conditions. Cells were stimulated with LPS/IL-6/TNF-α to induce inflammation. The secretion levels of TNF-α and IL-1β were quantified and expressed as pg/mL. Under inflammatory conditions, cytokine production was markedly increased compared with non-induced controls. Treatment with both the gel extract and AV: SF mixture significantly reduced TNF-α and IL-1β secretion in both cell types. Data are presented as mean ± SD. Different letters (a–d) indicate statistically significant differences between groups (* *p* < 0.05), where a* represents differences between TNF-α-induced and non-induced conditions, b* represents differences between treatment and non-treatment under TNF-α-induced conditions, c* represents differences between IL-1β-induced and non-induced conditions, and d* represents differences between treatment and non-treatment under IL-1β-induced conditions.

Under basal conditions, both THP-1 cells and HDdF secreted low levels of TNF-α and IL-1β, and neither the AV: SF mixture nor the gel extract significantly altered cytokine production. In contrast, inflammatory stimulation markedly increased the secretion of both cytokines in the two cell types (Figure 6).

In THP-1 cells (Figure 6a), TNF-α secretion was significantly elevated following inflammatory stimulation compared with the non-induced control. Treatment with the gel extract and the AV: SF mixture reduced TNF-α secretion by 39.60% and 43.58%, respectively (Table 1). Similarly, IL-1β secretion decreased by 33.56% and 31.04%, respectively.

In HDdF (Figure 6b), inflammatory stimulation also increased TNF-α secretion, which was subsequently reduced by 23.53% following treatment with the gel extract and by 17.49% with the AV: SF mixture. Likewise, IL-1β secretion decreased by 53.82% and 71.37%, respectively.

The marked reduction in TNF-α and IL-1β secretion under inflammatory conditions indicates that both formulations effectively modulate excessive inflammatory responses while preserving basal cytokine production. This selective anti-inflammatory activity is consistent with the complementary biological functions of the gel components. *Aloe vera* contains bioactive polysaccharides and phenolic compounds that suppress inflammatory mediator production and regulate inflammatory signaling pathways [10,16,17,18,37], whereas silk fibroin provides an extracellular matrix-like microenvironment that supports tissue repair while attenuating inflammatory responses [9,12,15,17,23,37]. Chitosan may further enhance these effects through its intrinsic immunomodulatory properties and by stabilizing the thermoresponsive gel matrix, thereby prolonging the retention and sustained release of the incorporated bioactive compounds [20,21,22,26].

The comparable inhibitory effects observed for the AV: SF mixture and the gel formulation suggest that the anti-inflammatory activity primarily arises from the intrinsic biological properties of *Aloe vera* and silk fibroin. Nevertheless, incorporation into the thermoresponsive gel provides additional advantages by maintaining prolonged contact with the wound surface and sustaining local bioactive availability, which may further improve therapeutic efficacy during chronic wound healing [13,14,20,21,22,26,27].

Collectively, these findings demonstrate that the AV: SF system effectively suppresses the secretion of the major pro-inflammatory cytokines TNF-α and IL-1β under inflammatory conditions while preserving basal cytokine production. This selective immunomodulatory activity is consistent with previous reports demonstrating that bioactive gels regulate macrophage-mediated inflammation and promote tissue repair [1,4,17,32,33,34,35,37]. Furthermore, these findings provide a mechanistic basis for the subsequent investigation of intracellular inflammatory signaling pathways, particularly NF-κB activation.

### 2.5. NF-κB Pathway

**Figure 7:**
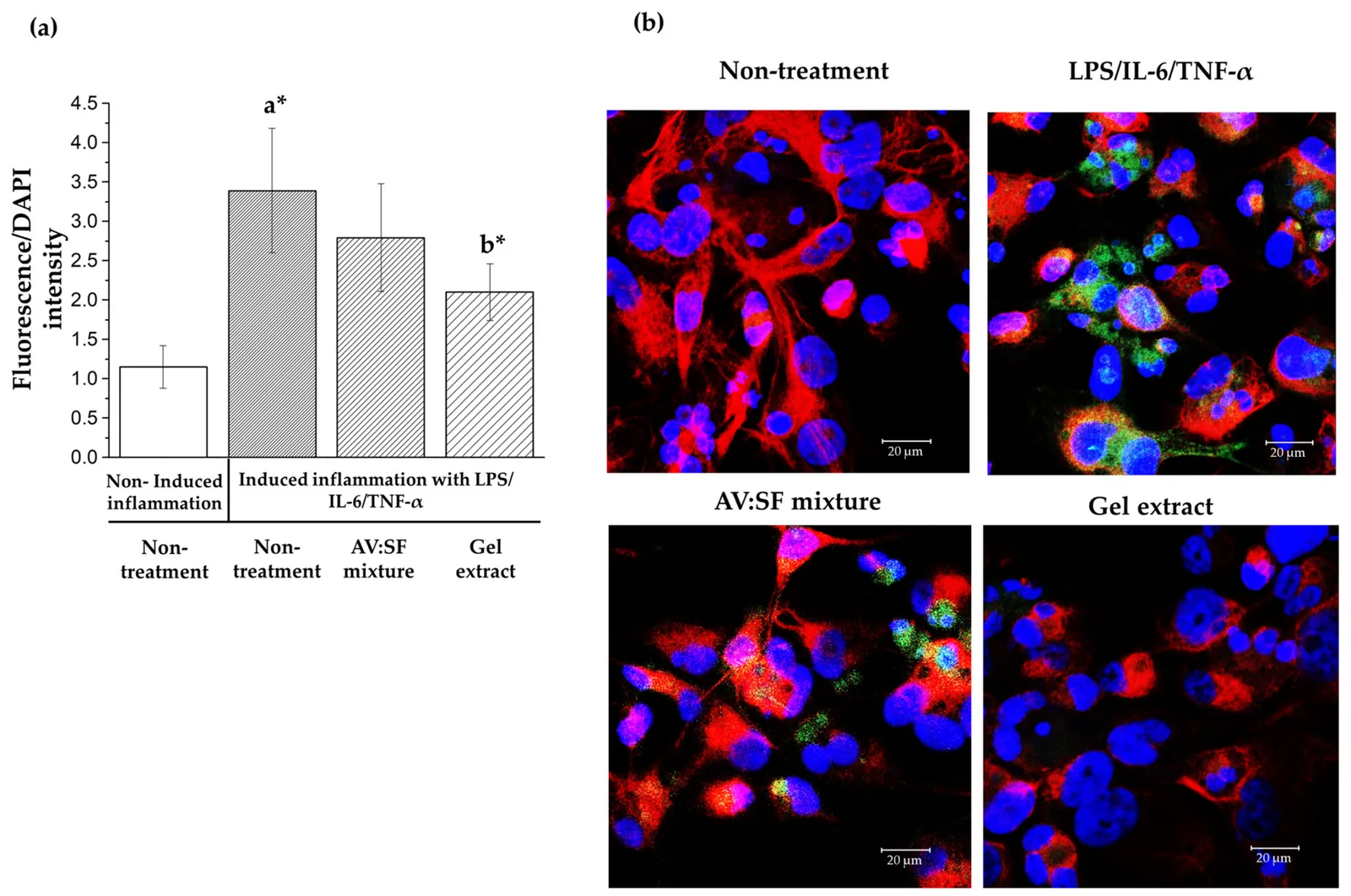
NF-κB p65 and CD68 immunofluorescence in macrophage-like THP-1 cells. CD68 (red) marks M0 macrophages, NF-κB p65 (green) indicates pathway activation, and nuclei are stained with DAPI (blue). (**a**) Quantification of NF-κB p65 fluorescence intensity (green/DAPI ratio) in control, extract-treated, and gel-treated groups (**b**) Representative immonofluoresence imaged showing CD68 (red), NF-κB p65 (green), and DAPI-stained nuclei (blue) under inflammatory stimulation (LPS/IL-6/TNF-α) conditions. Scale bar = 20 µm. Statistical significance was determined using one-way ANOVA followed by Fisher’s LSD post hoc test. Different letters indicate statistically significant differences (* *p* < 0.05) between groups, where “a” represents differences between induced inflammation and non-induced control, and “b” represents differences between treatment and non-treatment under induced inflammatory conditions.

**Figure 8:**
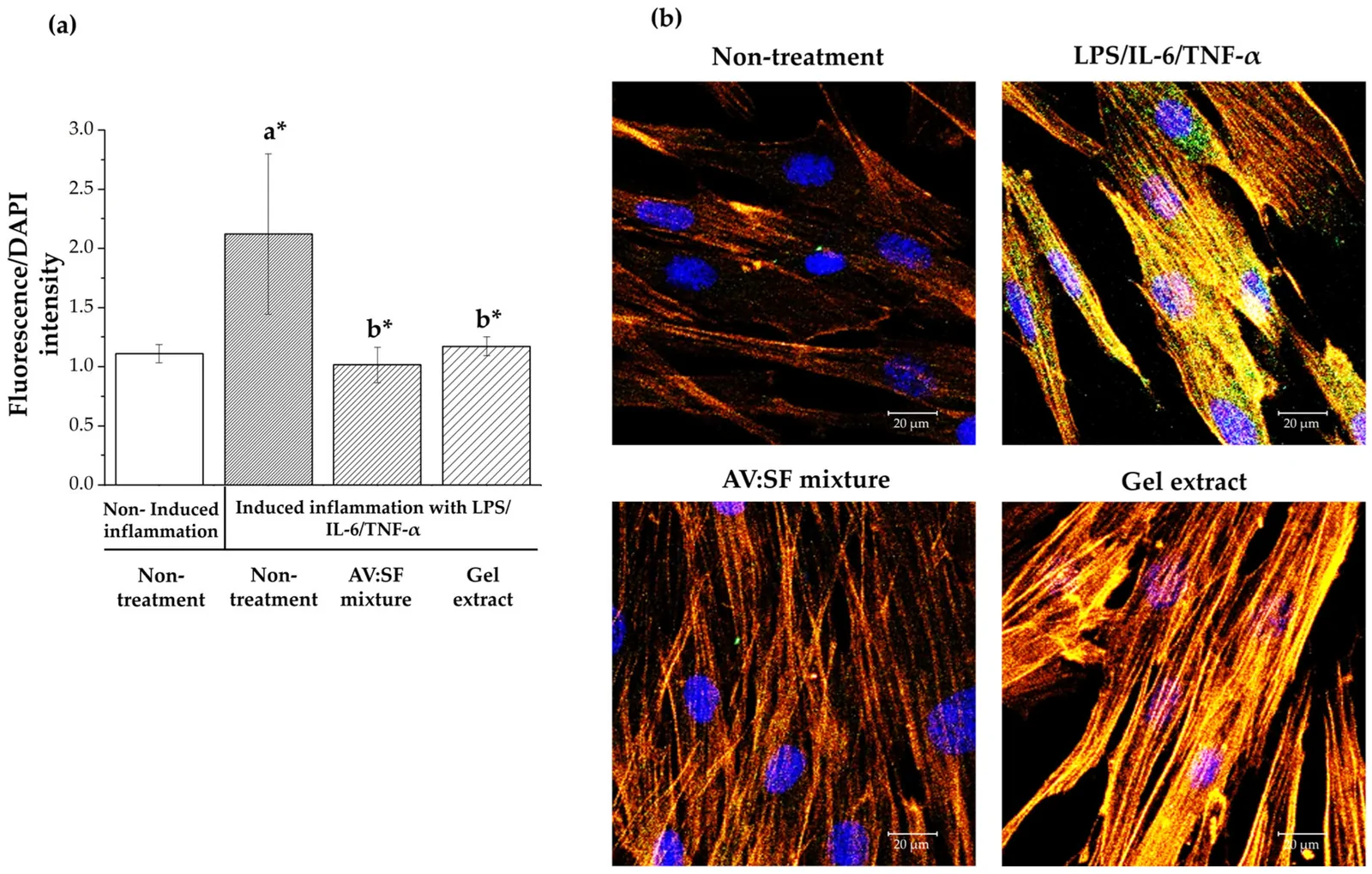
NF-κB p65 immunofluorescence in human diabetic dermal fibroblast (HDdF) cells. NF-κB p65 (green) indicates pathway activation, F-actin (red/orange) represents cytoskeletal organization, and nuclei are stained with DAPI (blue). (**a**) Quantification of NF-κB p65 fluorescence intensity (green/blue ratio) under non-induced, inflammatory (LPS/IL-6/TNF-α), AV:SF mixture-treated, and gel-treated conditions. (**b**) Representative immunofluorescence images showing NF-κB p65 (green), F-actin (red/orange), and DAPI-stained nuclei (blue) under non-induced, inflammatory (LPS/IL-6/TNF-α), AV:SF mixture-treated, and gel-treated conditions. Scale bar = 20 µm. Statistical significance was determined using one-way ANOVA followed by Fisher’s LSD post hoc test. Different letters indicate statistically significant differences (* *p* < 0.05) between groups, where “a” represents differences between induced inflammation and non-induced control, and “b” represents differences between treatment and non-treatment under induced inflammatory conditions.

The effects of the AV: SF mixture and gel extract on NF-κB signaling were evaluated in macrophage-like THP-1 cells and human diabetic dermal fibroblasts (HDdF) under non-induced and inflammatory conditions. An in vitro inflammatory model was established using combined stimulation with lipopolysaccharide (LPS), interleukin-6 (IL-6), and tumor necrosis factor-alpha (TNF-α) to reproduce the pro-inflammatory microenvironment associated with chronic diabetic wounds [28,29,30,31]. NF-κB p65 activation was evaluated by immunofluorescence staining based on fluorescence intensity and intracellular localization (Figure 7 and Figure 8).

Macrophage-like THP-1 cells were co-stained with CD68 (red) to confirm macrophage identity. CD68 expression remained stable across all experimental groups, indicating that neither inflammatory stimulation nor treatment altered macrophage identity. Under non-induced conditions, NF-κB p65 (green) exhibited weak fluorescence and was predominantly localized in the cytoplasm in both THP-1 cells and HDdF, indicating an inactive basal state. Following inflammatory stimulation, NF-κB p65 fluorescence intensity markedly increased and was accompanied by pronounced nuclear translocation in both cell types, confirming activation of the NF-κB signaling pathway [10,28,29,30,31].

Treatment with both the AV: SF mixture and gel extract significantly reduced NF-κB p65 fluorescence intensity in THP-1 cells under inflammatory conditions (Figure 7a, *p* < 0.05), with the gel extract producing a greater inhibitory effect than the AV: SF mixture. In HDdF, both formulations also significantly decreased NF-κB p65 fluorescence intensity and nuclear translocation compared with the inflammatory control (Figure 8a, *p* < 0.05), although no significant difference was observed between the two treatments. Representative immunofluorescence images further demonstrated reduced nuclear accumulation of NF-κB p65 after treatment. In addition, inflammatory stimulation disrupted F-actin organization, whereas both formulations partially restored cytoskeletal architecture.

The inhibition of NF-κB activation is consistent with the reductions in intracellular ROS and the secretion of TNF-α and IL-1β observed in the preceding experiments, suggesting coordinated regulation of oxidative stress and inflammatory signaling. *Aloe vera* contains bioactive polysaccharides and phenolic compounds that suppress NF-κB activation and inflammatory mediator production [10,16,17,18,37], whereas silk fibroin provides an extracellular matrix-like microenvironment that supports tissue repair while attenuating inflammatory responses [9,12,15,17,23,37]. Chitosan may further enhance these effects through its intrinsic immunomodulatory properties and by stabilizing the thermoresponsive gel matrix, thereby improving the retention and sustained release of the incorporated bioactive compounds [19,20,21,22,26,27].

Although both formulations effectively inhibited NF-κB activation, the gel extract generally produced a stronger response in macrophage-like THP-1 cells, suggesting that the thermoresponsive gel matrix enhances the biological activity of the incorporated bioactive compounds through prolonged local retention and sustained release. Collectively, these findings demonstrate that the AV: SF thermoresponsive gel effectively attenuates NF-κB activation while preserving basal signaling under non-induced conditions. These results provide mechanistic evidence supporting its antioxidant and anti-inflammatory activities and further support its potential application as a multifunctional biomaterial for chronic diabetic wound healing [1,4,17,32,33,34,35,37].

### 2.6. Effect of Gel Formulation and Extracts on Macrophage Polarization

**Figure 9:**
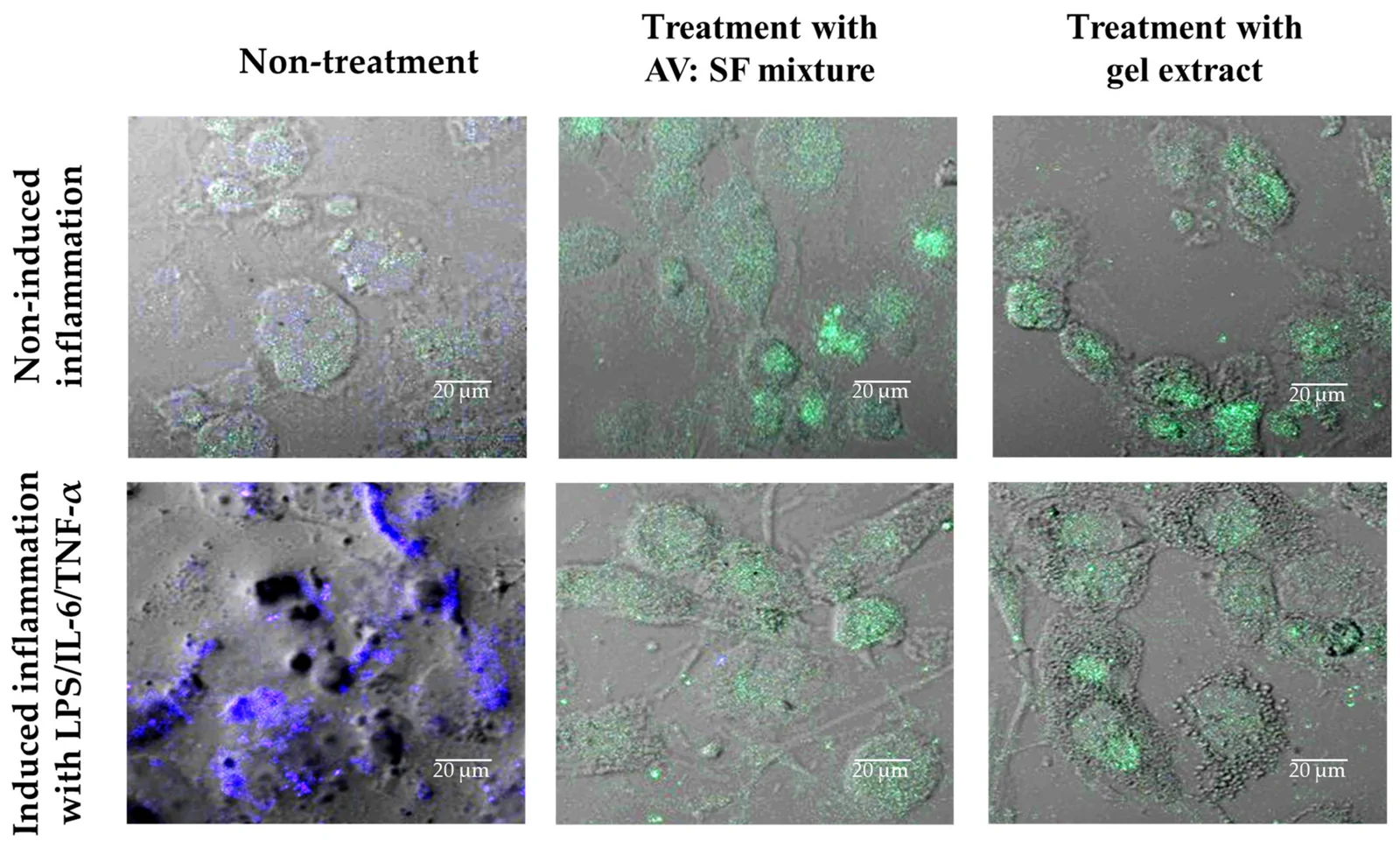
Representative fluorescence images of macrophage-like THP-1 cells showing CD80 (blue) and CD206 (green) expression under different conditions, including untreated control, inflammatory stimulation (LPS/IL-6/TNF-α), and treatment with the AV: SF mixture and gel extract under inflammatory conditions. Scale bar = 20 µm.

An in vitro inflammatory model was established using combined stimulation with lipopolysaccharide (LPS), interleukin-6 (IL-6), and tumor necrosis factor-alpha (TNF-α) to reproduce the pro-inflammatory microenvironment associated with chronic diabetic wounds [28,29,30,31]. The effects of the AV: SF mixture and gel extract on macrophage polarization were evaluated in macrophage-like THP-1 cells using CD80 as a marker of the pro-inflammatory M1 phenotype and CD206 as a marker of the anti-inflammatory M2 phenotype. The expression of both markers was examined by immunofluorescence staining (Figure 9).

Following inflammatory stimulation, macrophage-like THP-1 cells exhibited increased CD80 immunofluorescence intensity compared with the non-stimulated control, suggesting a shift toward an M1-like inflammatory phenotype based on qualitative imaging observations.

Treatment with both the AV: SF mixture and gel extract visibly reduced CD80 staining while enhancing CD206 immunofluorescence relative to the stimulated control, indicating a tendency toward an M2-like phenotype.

Because these observations were derived from qualitative immunofluorescence imaging, they should be interpreted as indicative evidence of phenotypic modulation rather than quantitative confirmation of macrophage polarization.

Macrophage phenotype plays a pivotal role in regulating wound healing. During the early inflammatory phase, M1 macrophages eliminate invading pathogens and damaged tissue through the production of pro-inflammatory mediators. However, persistent M1 activation is a hallmark of chronic diabetic wounds and contributes to sustained inflammation, impaired tissue repair, and delayed healing. In contrast, M2 macrophages facilitate the resolution of inflammation, extracellular matrix remodeling, angiogenesis, and tissue regeneration through the secretion of anti-inflammatory and pro-regenerative mediators [4,32,33,34,35]. Accordingly, therapeutic strategies capable of promoting the transition from an M1- to an M2-like phenotype are considered beneficial for restoring normal wound healing.

The qualitative changes observed in CD80 and CD206 expression are consistent with the reductions in intracellular ROS, TNF-α, IL-1β, and NF-κB activation demonstrated in the preceding experiments, suggesting that the AV: SF system regulates multiple components of the inflammatory cascade while promoting a pro-regenerative immune microenvironment. The similar responses observed for the AV: SF mixture and the thermoresponsive gel further indicate that the immunomodulatory activity primarily arises from the intrinsic biological properties of *Aloe vera* and silk fibroin [10,12,15,16,17,23,37], whereas the thermoresponsive gel matrix may further enhance therapeutic performance by improving local retention and sustaining the availability of the incorporated bioactive compounds [13,20,21,22,26,27].

Collectively, these findings demonstrate that the AV: SF system modulates macrophage phenotype under inflammatory conditions and supports the transition toward a pro-regenerative immune microenvironment. Together with its antioxidant and anti-inflammatory activities, these immunomodulatory effects further support the potential of the AV: SF thermoresponsive gel as a multifunctional biomaterial for chronic diabetic wound healing [1,4,17,32,33,34,35,37].

## 3. Conclusions

In this study, a thermoresponsive gel incorporating *Aloe vera* (AV) extract and silk fibroin (SF) was successfully evaluated. The formulation at 1.0 mg/mL maintained high viability in both macrophage-like THP-1 cells and human diabetic dermal fibroblasts (HDdF), indicating good biocompatibility under the tested in vitro conditions.

Overall, the AV:SF-based system demonstrated antioxidant and anti-inflammatory activities in an in vitro inflammatory diabetic-like model, as evidenced by reduced NF-κB signaling, decreased intracellular ROS levels, modulation of macrophage polarization markers (based on qualitative immunofluorescence analysis of CD80 and CD206), and increased fibroblast proliferation. These results indicate a potential ability of the formulation to regulate key cellular responses associated with impaired wound healing.

However, this study is limited to in vitro experiments and an inflammatory-induced wound-like model. Macrophage polarization was assessed only by qualitative immunofluorescence imaging without quantitative validation. In addition, angiogenesis, antimicrobial activity, cell migration, wound closure assays, co-culture systems, and in vivo diabetic wound models were not included.

Future studies should incorporate quantitative mechanistic analyses, functional wound healing assays, antibacterial evaluation, and in vivo validation to further confirm the therapeutic potential and translational relevance of this gel system.

## 4. Materials and Methods

### 4.1. Materials

Silk fibroin (SF) isolated from yellow silk cocoons (*Bombyx mori*) and *Aloe vera* gel extract (AV) isolated from fresh *Aloe vera* (L.) Burm.f. leaves were kindly provided by Professor Jarupa Viyoch, Biomaterial and Regenerative Medicine Research Unit, Faculty of Pharmaceutical Sciences, Naresuan University, Phitsanulok, Thailand. The protein molecular weight distribution and content were characterized as described in our previous reports.

Chitosan (CS, medium molecular weight; degree of deacetylation 80–95%) was purchased from Bio21 Co., Ltd. (Samut Sakhon, Thailand). Poloxamer 188 and 407, β-glycerophosphate (β-GP), polyhexamethylene biguanide (PHMB), phorbol 12-myristate 13-acetate (PMA), 2′,7′-dichlorodihydrofluorescein diacetate (DCFH-DA), thiazolyl blue tetrazolium bromide (MTT), and dimethyl sulfoxide (DMSO) were purchased from Sigma-Aldrich (St. Louis, MO, USA).

The human monocytic cell line THP-1 was obtained from the American Type Culture Collection (ATCC, Manassas, VA, USA). Human diabetic dermal fibroblasts (HDdF) were purchased from PELO Biotech GmbH (Planegg-Martinsried, Germany). RPMI-1640 medium, Dulbecco’s modified Eagle’s medium (DMEM), fetal bovine serum (FBS), penicillin–streptomycin (PS), trypsin-EDTA solution, and phosphate-buffered saline (PBS) were obtained from commercial suppliers. All other reagents were of analytical grade and used as received.

Mouse anti-human CD68 and rabbit anti-human NF-κB p65 antibodies were purchased from Abcam (Cambridge, MA, USA) and Cell Signaling Technology (Danvers, MA, USA), respectively. Goat anti-mouse Alexa Fluor 568 and goat anti-rabbit Alexa Fluor 488 antibodies were purchased from Thermo Fisher Scientific (Waltham, MA, USA). Mouse anti-human CD80 conjugated with BV421 and mouse anti-human CD206 conjugated with BB515 were purchased from Sony Biotechnology (San Jose, CA, USA) and BD Biosciences (San Jose, CA, USA), respectively.

### 4.2. Gel Preparation

The *Aloe vera*–silk fibroin–chitosan thermoresponsive gel was prepared according to our previous study [24] with slight modifications. Briefly, chitosan (CS) and a Poloxamer 188/407 blend were dissolved in 0.5 M lactic acid to form the polymeric base, and the pH was adjusted using 10% sodium bicarbonate solution. In parallel, *Aloe vera* extract (AV) and silk fibroin (SF) were dissolved in deionized (DI) water together with β-glycerophosphate (β-GP) and polyhexamethylene biguanide (PHMB). The two solutions were then combined and stirred until a homogeneous mixture was obtained.

The resulting formulation was stored under refrigerated conditions, during which thermoresponsive gelation occurred within 24–48 h, yielding a stable gel system. The AV: SF: CS ratio was fixed at 1:36:155, with a total polymer concentration of 4% *w*/*w*. The Poloxamer system, consisting of Poloxamer 188 and Poloxamer 407 at a ratio of 20:80, was used at a total concentration of 15% *w*/*w*. β-GP and PHMB were incorporated at concentrations of 2% and 0.1% *w*/*w*, respectively. The final pH of the gel was adjusted to 5.5–7.0.

### 4.3. Sample Preparation for Biological Activity Testing

For in vitro evaluation of the inflammatory response, the prepared gel was extracted in serum-free culture medium at 37 °C for 24 h under sterile conditions. The resulting extract was collected, filtered through a 0.22 µm membrane, and further diluted with culture medium to the desired concentrations for biological assays.

To assess the contribution of the bioactive components within the formulation, cells were also treated with an AV: SF (1:36) solution at a concentration equivalent to that present in the gel extract. The AV: SF mixture was prepared by dissolving the components in serum-free culture medium and incubating at 37 °C for 24 h under sterile conditions, followed by filtration through a 0.22 µm membrane before use.

### 4.4. Inflammatory Induction

THP-1 cells (2.5 × 10^5^ cells/well) were maintained in RPMI-1640 medium supplemented with 10% fetal bovine serum (FBS) and 1% penicillin–streptomycin (PS) at 37 °C in a humidified atmosphere containing 5% CO_2_. For differentiation into macrophage-like cells, THP-1 cells were seeded in 96-well plates and treated with 100 ng/mL phorbol 12-myristate 13-acetate (PMA) for 72 h, followed by a resting period in fresh medium to allow stabilization of the macrophage-like phenotype.

HDdF were seeded at 1 × 10^4^ cells/well in 96-well plates and cultured in DMEM supplemented with 5% FBS and 1% PS at 37 °C in a humidified atmosphere containing 5% CO_2_.

For induction of inflammatory conditions, macrophage-like THP-1 cells and HDdF were stimulated with 1 µg/mL lipopolysaccharide (LPS), 20 ng/mL interleukin-6 (IL-6), and/or 20 ng/mL tumor necrosis factor-alpha (TNF-α) in high-glucose (25 mM) culture medium for 24 h before treatment with either the gel extract or the AV: SF mixture.

### 4.5. Cytotoxicity of Macrophage-like THP-1 Cells, and HDdF

Macrophage-like THP-1 cells and HDdF under normal and inflammatory conditions were treated with various concentrations of gel extract according to our previous study, and cell viability was assessed using the MTT assay. Briefly, following 24 h of inflammatory stimulation, the medium was replaced with gel extract-containing medium and incubated for an additional 24 h. Subsequently, MTT solution (0.5 mg/mL) was added to each well and incubated for 4 h at 37 °C. The resulting formazan crystals were dissolved in dimethyl sulfoxide (DMSO), and absorbance was measured at 570 nm using a microplate reader (BioTek Synergy HTX, Winooski, VT, USA).

Under normal conditions, cells were cultured in standard medium without inflammatory stimulation. Cell viability was expressed as a percentage relative to untreated control cells. All experiments were performed in triplicate.

### 4.6. Proliferation of HDdF

Cell proliferation of the test samples was evaluated using the BrdU assay on HDdF under both normal and inflammatory conditions. Cells were seeded at 1 × 10^4^ cells/well in 96-well plates and incubated for 24 h at 37 °C in 5% CO_2_. Following this, the medium was replaced with either normal culture medium (DMEM supplemented with 5% FBS and 1%PS) or inflammatory medium containing LPS (1 µg/mL), IL-6 (20 ng/mL), and TNF-α (20 ng/mL), and cells were incubated for 24 h.

Subsequently, the medium was replaced with fresh medium containing gel extract at a concentration of 1.0 mg gel/mL DMEM (5% FBS, 1%PS), and cells were further incubated for 24 or 48 h. Cell proliferation was then assessed using a BrdU incorporation assay. Briefly, BrdU labeling solution was added and incubated for 24 h at 37 °C to allow incorporation into proliferating cells. After incubation, cells were processed according to the manufacturer’s protocol, and absorbance was measured at 570 nm using a microplate reader.

Cell proliferation was expressed as a percentage relative to untreated control cells. All experiments were performed in triplicate.

### 4.7. Reactive Oxygen Species (ROS) Assay

Intracellular ROS generation was assessed using the DCFH-DA assay (Sigma-Aldrich, St. Louis, MO, USA) in macrophage-like THP-1 cells and HDdF under both normal and inflammatory conditions, following the same cell culture procedures described above. Cells were treated with various concentrations of gel extract or AV: SF (1:36), at a concentration equivalent to that present in the gel extract, for 24 h before ROS measurement.

After treatment, cells were incubated with 10 µM DCFH-DA for 45 min at 37 °C in a humidified atmosphere containing 5% CO_2_. Cells were then washed with PBS to remove excess probe and stained with 4′,6-diamidino-2-phenylindole (DAPI) for 10 min at room temperature. All experiments were performed in triplicate.

Fluorescence intensity was measured using a BioTek SYNERGY H1 microplate reader with excitation and emission wavelengths of 485 nm and 532 nm, respectively. Intracellular ROS levels were expressed as a percentage relative to the non-induced, untreated control group.

Representative fluorescence images were acquired using a Thermo Fisher EVOS M3000 Digital Imaging System (Waltham, MA, USA). Fluorescence intensity was quantified as the ratio of DCF fluorescence to DAPI intensity.

### 4.8. TNF-α and IL–1β Secretion by ELISA

The secretion of pro-inflammatory cytokines, interleukin-1β (IL-1β) and tumor necrosis factor-alpha (TNF-α), was quantified using enzyme-linked immunosorbent assay (ELISA) in macrophage-like THP-1 cells HDdF under both normal and inflammatory conditions.

Cell culture supernatants were collected and centrifuged to remove cellular debris. The concentrations of TNF-α and IL-1β in the supernatants were measured using commercial ELISA kits (Medix Biochemica, Besançon, France) according to the manufacturer’s instructions.

Absorbance was recorded at 450 nm using a microplate reader. Cytokine concentrations were calculated from standard calibration curves and expressed as a percentage relative to the untreated inflammatory control group. All experiments were performed in triplicate.

### 4.9. NF-κB Pathway in Macrophages and HDdF by Immunofluorescence

The effect of the gel extract and AV: SF mixture on NF-κB signaling pathway activation was evaluated in macrophage-like THP-1 cells by immunofluorescent detection of the NF-κB p65 subunit together with the macrophage marker CD68. Cells were seeded at 2.5 × 10^5^ cells/well on 8-well coverslips and differentiated with PMA for 3 days at 37 °C in a humidified atmosphere containing 5% CO_2_. Cells were then induced into inflammatory conditions and subsequently treated with either the gel extract or the AV: SF mixture.

After treatment, THP-1 cells were washed with PBS, fixed with 4% (*w*/*v*) paraformaldehyde for 15 min, and permeabilized with 0.1% (*v*/*v*) Triton X-100 for 10 min. Non-specific binding sites were blocked using 3% (*v*/*v*) goat serum in PBS for 1 h at room temperature. Cells were then incubated overnight at 4 °C with primary antibodies against CD68 and NF-κB p65, diluted in blocking solution. After washing with PBS, cells were incubated with goat anti-mouse Alexa Fluor 568 and goat anti-rabbit Alexa Fluor 488 secondary antibodies for 1 h at room temperature in the dark. Nuclei were counterstained with DAPI.

For HDdF, cells were washed with PBS, fixed with 4% (*w*/*v*) paraformaldehyde for 15 min, and permeabilized with 0.1% (*v*/*v*) Triton X-100 for 10 min. Non-specific binding was blocked using 5% (*v*/*v*) goat serum in PBS for 1 h at room temperature. Cells were incubated overnight at 4 °C with the primary antibody against NF-κB p65, diluted in blocking solution. After washing with PBS, cells were incubated with goat anti-rabbit Alexa Fluor 488 secondary antibody, while F-actin was stained using phalloidin-conjugated rhodamine for 1 h at room temperature in the dark. Nuclei were counterstained with DAPI.

Images were acquired using a ZEISS LSM 800 confocal fluorescence microscope (Oberkochen, Germany). Fluorescence intensities were analyzed by Zen Blue 3.11 software.

### 4.10. Macrophage Polarization by Immunofluorescence

M1 and M2 macrophage polarization was evaluated by immunofluorescence staining of CD80 (M1 marker) and CD206 (M2 marker) in macrophage-like THP-1 cells. Cells were seeded at 2.5 × 10^5^ cells/well on 8-well coverslips and incubated for 24 h at 37 °C in a humidified atmosphere containing 5% CO_2_. Subsequently, THP-1 cells were differentiated into macrophage-like cells using PMA for 3 days under the same culture conditions. Cells were then induced into inflammatory conditions and treated with either the gel extract or the AV: SF mixture.

After treatment, cells were washed with PBS and fixed with 4% (*w*/*v*) paraformaldehyde for 15 min at room temperature, followed by blocking with 3% (*w*/*v*) bovine serum albumin (BSA) in PBS for 1 h.

Cells were then incubated with CD80 and CD206 antibodies for 1 h at room temperature in the dark. Nuclei were counterstained with DAPI.

Images were captured using a ZEISS LSM 800 confocal fluorescence microscope.

### 4.11. Statistical Analysis

Data are presented as mean ± standard deviation (SD) from three independent experiments. Statistical significance was assessed using one-way analysis of variance (ANOVA) followed by Fisher’s LSD post hoc test at a 95% confidence level. Significance is indicated as *p* < 0.05, *p* < 0.01, and *p* < 0.001.

## 5. Patents

The gel formulation used in this study was prepared according to petty patent No. 2503002834, entitled “The process of gel production for diabetic wound healing incorporating silk fibroin and *Aloe vera* gel extract,” registered with the Department of Intellectual Property, Thailand (1 August 2025).

## Figures and Tables

**Table 1:** % Reduction in secretion treated with AV: SF mixture and gel extract compared to induced inflammation with LPS/IL-6/TNF-α.

Samples	Cell Types	% Reduction in Secretion	
		TNF-α	IL-1β
AV: SF mixture	Macrophage-like THP-1	43.58 ± 0.99	31.04 ± 1.32
	HDdF	17.49 ± 6.09	71.37 ± 5.15
Gel extract	Macrophage-like THP-1	39.60 ± 2.32	33.56 ± 1.58
	HDdF	23.53 ± 6.02	53.82 ± 7.52

## Data Availability

Data is contained within the article.

## Abbreviations

The following abbreviations are used in this manuscript:

LPS	Lipopolysaccharide
TNF-α	Tumor Necrosis Factor-alpha
IL-6	Interleukin–6
*w*/*v*	weight/volume
*w*/*w*	weight/weight
PHMB	Polyhexamethylene Biguanide
β–GP	β–glycerophosphate
RPMI	Roswell Park Memorial Institute medium
DMEM	Dulbecco’s Modified Eagle Medium
MTT	3–(4,5–dimethylthiazol–2–yl)–2,5–diphenyltetrazolium bromide
FTIR	Fourier Transform Infrared Spectroscopy
THP–1	Human monocytic leukemia cell line
HDbF	Human diabetic dermal fibroblast
CD68	Cluster of Differentiation 68
CD80	Cluster of Differentiation 80
CD208	Cluster of Differentiation 208
NF–kB	Nuclear Factor kappa-light-chain-enhancer of activated B cells
